# Academic Performance and the Link with Depressive Symptoms among Rural Han and Minority Chinese Adolescents

**DOI:** 10.3390/ijerph19106026

**Published:** 2022-05-16

**Authors:** Tianli Feng, Xiyuan Jia, Lucy Pappas, Xiaojun Zheng, Teresa Shao, Letao Sun, Charlie Weisberg, Madeline Lu Li, Scott Rozelle, Yue Ma

**Affiliations:** 1 School of Management and Economics, University of Electronic Science and Technology of China, Chengdu 610054, China; tianlie@uestc.edu.cn; 2 School of Public Administration, Northwest University, Xi’an 710069, China; 3 Stanford Center on China’s Economy and Institutions, Stanford University, Stanford, CA 94305, USA; lmpappas@stanford.edu (L.P.); teresa01px2022@saschina.org (T.S.); sun67301@gapps.uwcsea.edu.sg (L.S.); cweisberg23@groton.org (C.W.); rozelle@stanford.edu (S.R.); yma3@stanford.edu (Y.M.); 4 Suide Middle School, Yulin 718000, China; suideermao@163.com; 5 College of Letters & Science, University of California Santa Barbara, Santa Barbara, CA 93106, USA; l.l.madeline5678@gmail.com

**Keywords:** depressive symptoms, academic performance, China, rural Han students, rural minority students, children and adolescents

## Abstract

The objectives of this paper were to examine the risk of depression and depressive symptoms among Han and minority children and adolescents in rural China, the links between academic performance and depressive symptoms, and the prevalence of these links among specific subgroups. A total of 8392 4th, 5th, and 6th grade students at 105 sample rural schools in eight low-income counties and districts in a prefectural-level city in Southwestern China were randomly selected using a three-step sampling strategy. A total of 51% of the sample were female (SD = 0.50), and the age range was 7 to 19 years (mean = 11.35 years; SD = 1.05). Using the Patient Health Questionnaire 8-item depression scale, the prevalence of depressive symptoms in the sample was assessed, while data on students’ academic performance (standardized math test) and demographic characteristics were also collected. Our results show that the rates of major depression were 19% for Han students, 18% for Tibetan students, and 22% for Yi students; the rates of severe depression were 2% for Han and Tibetan students, and 3% for Yi students. Yi students were at significantly higher risks for major and severe depression than Han students. We conducted multivariate regression and heterogeneous analyses. Academic performance was negatively and significantly correlated to depressive symptoms. Across the whole sample, students with lower math scores, minority students, boys, younger students, and students with migrant parents were most vulnerable to depressive symptoms. The heterogeneous analysis suggests that among poor-performing students, subgroups at higher risk for depression include boys, non-boarding students, and students whose mothers had graduated from high school or above. These findings indicate a need to improve mental health outcomes of rural Han and minority primary school students, targeting academic performance for possible intervention.

## 1. Introduction

Child and adolescent depression in low- and middle-income countries (LMICs) is a serious public health concern, as up to 20% of children (3–10 years) and adolescents (10–19) in LMICs are affected by mental illness [1]. Internationally, depression is a leading cause of childhood and adolescent social, health, and psychological problems, which includes increased rates of suicidal thoughts and suicide attempts [2]. Furthermore, depression has long-lasting impacts, too, including increased levels of adult anxiety and substance abuse, as well as worse health, criminal, and social functioning [3]. Moreover, international research has reported significant associations between academic performance and the risk of depression [4,5,6], as academic stress has been linked to depression in adolescent students [4,5,7].

Consistent with the international literature, research from China, an LMIC, indicates that depression is common among children and adolescents throughout the country, in both rural and urban settings. High rates (~20%) of major depression among children and adolescents have been reported in numerous studies using the Children’s Depression Inventory (CDI), Center for Epidemiological Studies Depression Scale (CES-D), Zung’s Self-rating Depression Scale (SDS), and the Depression Self-Rating Scale for Children (DSRSC) [8,9,10,11]. For example, a meta-analysis of 62 studies on depressive symptoms in children and adolescents from rural and urban China reported that the overall pooled prevalence of depressive symptoms (measured by a range of instruments including SDS, CDI, CES-D, The Symptom Checklist subscales for depression, DSRSC, Beck Depression Inventory-II, Beck Depression Inventory BDI, and Psychological Health Inventory) was 22.2% [12]. Another systematic review and meta-analysis of 27 studies and 42,374 primary school students (aged 7–15 years) across China identified the pooled prevalence rate of depression to be 17.2% (measured by CDI, CES-D, DSRSC, and the BDI) [13]. However, evidence from 25 provinces in a longitudinal social survey (using CES-D) showed that the prevalence of childhood depression is higher in rural areas (23%) than in urban areas (14%), suggesting that children and adolescents living in rural regions may be at greater risk [8].

Additionally, consistent with international findings, academic performance has been shown to be linked with depression among children and adolescents in China. In China, where competitive entrance examinations are widely accepted as the norm, and education is highly valued by society, research shows that children and adolescents are likely to suffer from mental health problems related to their academic performance [7,14]. In fact, a study of more than 2000 adolescent students in urban and rural China found significant correlations between academic performance, measured by average grades over the past year, and depression, measured by CES-D [15]. In addition, research from rural China has found that poor academic performance (measured by standardized math tests and academic grades) is correlated to increased feelings of anxiety and depression (measured by Mental Health Test MHT, the Revised Children’s Manifest Anxiety Scale, Fear Survey Schedule for Children-Revised, and BDI) among primary school students [16,17].

Due to the severity of childhood and adolescent depression in China, more research should examine what characteristics make the youth more and less vulnerable to depression. Studies in China have shown that specific subgroups are affected by depression differently, including girls, students of migrant parents, and boarding students. According to one study from rural Taiwan, poor-performing female elementary school students reported higher risks of depression (measured by CES-D) than poor-performing male peers [7]. Gao et al. [7] also found that poor-performing students, whose academic performance was measured through a standardized math test, were at greater risk of depression if their fathers migrated from home, but a lower risk of depression if their mothers had migrated. Research examining the effect of school boarding on mental health has mixed results. Chen et al. [18] identified negative association between school boarding and student mental health (Children’s Manifest Anxiety Scale) among elementary school students in rural western China; however, Tang et al. [19] found no significant associations in a sample from the same area with similarly aged students, measuring depressive symptoms with a self-designed MHT. To the best of our knowledge, there are no studies that examine the effects of parent educational attainment on depressive symptoms among the Chinese youth, although international findings suggest that more highly educated parents spend more time with their children than less-educated parents [20,21,22]. Furthermore, evidence from Taiwan has found that youths with lower levels of parental care or engagement are more likely to be depressed (measured by the MHT) [23]. In addition, while there is no data from China, the international literature suggests a link between being an only child and depressive symptoms, as several studies identified that adolescents who are only children demonstrate better mental health (measured by the Strengths and Difficulties Questionnaire) than those with siblings [24,25]. Despite this research, it remains unclear how the influence of academic performance on depressive symptoms in rural China varies by ethnicity, gender, age, being an only child, boarding status, parent educational attainment, and parent migration status.

In China, several vulnerable subgroups have been neglected in past studies on childhood and adolescent depression, specifically rural and minority groups. Out of the 1.4 billion people who live in China, more than half (55%) of the population, including 250 million children (0–14 years old), are considered rural, which is determined by legal residency status. (Legal residency status refers to China’s household registration system (*hukou*). The system, which began in the late 1950s, classifies the residency status of all individuals as either rural or urban, with social benefits determined accordingly, with urban *hukou* providing more privileges than rural *hukou*. In addition, *hukou* identifies individuals by their “places of origin” in China [26].) In addition, despite comprising a small share of the total population, minority ethnic groups are an important part of China’s national body. The Han ethnicity is the majority ethnic group, representing approximately 91% of the total population. The other 9% is comprised of non-Han minority groups, including Yi and Tibetan ethnic groups, for example [26]. To the best of our knowledge, there is a large gap in research investigating the links between academic performance and mental health in rural minority and rural Han groups. Based on data from 2012 from a sample of 2679 children from rural western China, Zhou et al. [8] found that minority children are significantly more likely to suffer from depression than Han children. While that study focused on studying the prevalence of child depression in a rural and minority sample, there remains a gap in the literature for more recent data on the links between academic performance and depression in a large rural and minority sample.

The goal of this study was to document the prevalence of depression among rural Han and minority primary school students in western China (aged 7–19 years) and explore the links (and the nature of the links) between academic performance and depression. First, we reported the rate of depression among rural primary school students in a province in western China and compared the depressive symptom rates of Han and minority students. Next, correlation between academic performance and depressive symptoms were measured throughout the whole sample and ethnic group subsamples. Finally, we investigated results from heterogeneous analyses to determine which subgroups—including minorities, girls, boarding students, only children, students whose parents migrated away for work, and students whose parents had not completed high school—reported the strongest correlations between academic performance and depression.

## 2. Materials and Methods

### 2.1. Ethical Approval

Ethical approval for this study was granted by the Stanford University Institutional Review Board (IRB No. 35635). All subjects gave written informed consent in accordance with the Declaration of Helsinki. All subjects were free to choose whether they were willing to participate in the research.

### 2.2. Sampling Selection

The data were collected in the spring of 2021 from eight counties and districts in City A, in Province A (Southwestern China). All eight counties and districts have relatively low levels of economic development. For example, compared to the other prefecture-level cities in Province A, the GDP per capita for City A (46,984 RMB; 7265 USD) is below the mean GDP per capita for all other cities in Province A (47,729 RMB; 7404 USD) [27]. Furthermore, the majority of City A residents (55%) are rural and have considerably lower income levels than urban residents. The per capita disposable income of City A rural residents in 2019 (14,586 RMB; 2256 USD) was one of the lowest when compared to other prefecture-level cities in Province A and was less than half the disposable income of City A’s urban residents (35,043 RMB; 5419 USD) [27]. In terms of demographics, City A is home to both Han and minority populations, and has a total population count of approximately 1.5 million [27]. Regarding the school system, official statistics report that there are a total of 161 primary schools in the city, and a total of 85,349 primary school students [27].

Within City A, we used a three-step sampling strategy to select our study sample. First, the local education bureau of each county or municipality directly under the central government provided our team with the names of all primary schools within City A. From the list, all non-six-year schools were excluded from the study. In total, 105 schools were randomly selected to be included in the study. Second, in all 105 sample schools, one class from each grade (4th grade, 5th grade, and 6th grade) was randomly chosen. Finally, all the students in each of the sample classes were included in our study, which totaled 8647 students. Due to each school reporting several students who were unable to finish the survey form, 255 students were not included in the final sample. A total of 8392 students from 4th, 5th, and 6th grades participated in the study. From the final sample, the average number of students in a given county was 1235 with a range of 355 to 1616 students, the average school size was 82 students with a range of 22–181 students, and the average class size was 27 students with a range of 3 to 52 students.

### 2.3. Data Collection

In March 2021, at the beginning of the second semester of the 2020–2021 academic year, a research team composed of local college students conducted a three-part survey that collected three blocks of data: (a) measures of student depressive symptoms, (b) measures of student academic performance, and (c) student demographic characteristics. Before administering the survey in the sample schools, the members of the research team completed several days of survey training from the leaders of the research team. In each sample school, the research team followed strict protocols in managing the survey process, which included giving detailed explanations of the survey questions to sample classes and enforcing strict time limits during surveying.

#### 2.3.1. Depressive Symptoms

The first survey block used the Patient Health Questionnaire 8-item depression scale (PHQ-8) to measure depressive symptoms of sample students. The PHQ series is one of the most widely used assessment methods for measuring symptoms of depression in the world (e.g., [28,29,30]. International literature confirms the PHQ series as an appropriate tool for assessing the risk of depression among the general public, children, and adolescents [29,31,32,33]. The test has been translated into the Chinese language, and its reliability and validity have been verified inside China [34,35]. Although the PHQ-8 series has not been previously used to evaluate depressive symptoms of primary school students in China, this assessment has been used to evaluate depressive symptoms of college and medical students in China [19,34,36,37]. Moreover, the PHQ-9 was validated and proven reliable in Mandarin Chinese [38] and has been validated as an effective self-report scale for screening depression in children and adolescents in China [39,40,41]. Given that the PHQ-8 and PHQ-9 instruments differ by only one survey item (a question regarding suicide and self-harm ideation, which can be a sensitive subject for younger participants), the PHQ-8 was used in this study.

The PHQ-8 is a self-assessment of eight items that are scored on a 4-point Likert scale. For each item there are four possible answers (scaled 0 to 3) that correspond to a frequency of experiencing a certain emotion or taking a certain action in the past two weeks: 0 = “not at all” (0 to 1 day), 1 = “several days” (2 to 6 days), 2 = “more than half the days” (7 to 11 days), or 3 = “nearly every day” (12 to 14 days). For the final score, the scores from all eight items are summed to calculate a score between 0 and 24 points. Mean scores for the sample and subgroups are then calculated. To determine the level of risk of depression, we used the cut-off scores that have been commonly used in the literature [29]. We based score cutoffs on previous research that defined a score of 10 or higher as risk of “major depression” (defined as any type of moderate, moderately severe, or severe depression) and a score of 20 or higher as risk of “severe depression” [29].

#### 2.3.2. Academic Performance

For the second survey block, a standardized math test was used to measure the academic performance of the sample students. Standardized math exams have been used widely in previous high-quality research papers in China to measure academic performance as an outcome variable [42,43,44,45,46]. In our study, the academic performance outcome is based on student math score, which is measured during the survey using a 35-min-long math test. The math test was designed by trained psychometricians, with the math test items for the endline and baseline tests selected from the standardized mathematics curricula for primary school students in China. To confirm our test was reliable, the content validity of these test items was checked by multiple experts. The psychometric properties of the tests were then validated using data from extensive pilot testing to ensure good distributional properties (no bottom or top coding, for instance). In the analyses, we normalized mathematics performance scores.

The research team collaborated with the local education bureau and primary school teachers to design a math test based entirely on the mathematics curriculum of China’s primary schools. However, to eliminate the risk of bias from teachers who might prepare students for the test in advance, test design was limited to teachers whose students were not participating in the survey. Students were given 35 min to complete the math test (which included 35 items), throughout which the research team closely monitored the sample students on time and to avoid cheating. After students completed the test, the scores were standardized for analysis. Standardizing was completed by scaling raw scores into z-scores, which were calculated by subtracting the mean score from the raw score, then dividing the difference by the standard deviation (SD) of the distribution of scores from all students in each of the three grades.

#### 2.3.3. Demographic Characteristics

In the third and final survey block, a questionnaire asking about students’ individual and household characteristics was used to measure demographic characteristics. The questionnaire asked each student to report their ethnicity, gender, age, whether they were an only child, and whether they were a boarding student. Additionally, students were asked whether their parents lived at home for the majority of the past previous semester, and about the educational attainment status of their parents. Lastly, to assess household income, students were asked whether their households had access to a computer with Internet, refrigerator, air conditioner, microwave, and car. These answers were used to construct an asset-based wealth index using the principal component analysis approach (PCA) [47].

### 2.4. Statistical Analysis

The statistical analysis consisted of three parts. First, to examine the prevalence of depression overall and across subgroups, we compared measures of depressive symptoms across six levels of depression: mild depression, moderate depression, moderately severe depression, severe depression, major depression, and the average depression score. We compared the measures of depressive symptoms across subgroups which were determined by the individual and family characteristics of the sample students. The subgroups were determined by (a) math score; (b) student ethnicity; (c) student gender; (d) student age; (e) only child status; (f) school boarding status; (g) whether the student’s parents lived at home last semester; (h) the educational attainment of the parents of the students; and (i) household asset value. Independent sample *t*-tests were used to measure significant differences in levels of depressive symptoms between Han and Tibetan subsamples, and Han and Yi subsamples, and to analyze which individual and household characteristics correlated to depression.

Second, to estimate conditional correlation between academic performance and depression, we used the following function for multivariate analysis with an added vector of control variables:

(1) $$Y_{i}=\alpha +\beta_{1}score_{i}+\gamma x_{i}+\Phi_{c}+\epsilon_{i},$$

where the dependent variable $Y_{i}$ represents the depression level of student i. The dependent variable $Y_{i}$ has different measures of depression level, including risk of mild depression (1 if 9 ≥ score ≥ 5); risk of moderate depression (1 if 14 ≥ score ≥ 10); risk of moderately severe depression (1 if 19 ≥ score ≥ 15); risk of severe depression (1 if score ≥ 20); and risk of major depression (1 if score ≥ 10). We estimated Equation (1) using the student’s total depression score as the dependent variable ($Y_{i}$). 

Beyond the dependent variable, the right-hand side variables included several different values. Score indicates the standardized math score. The vector $x_{i}$ indicates students’ individual and household characteristics. The individual characteristics include whether the student belongs to a minority group (1 = student is Han), student gender (1 = female), student age (in years), only child status (1 = student is an only child), and boarding student status (1 = student is boarding). The household characteristics include whether the parent is a migrant worker (1 = father/mother of the student did not live at home with the student for the majority of the past semester), the educational attainment of parents (1 = father/mother of the student graduated from high school or above), and the household asset value (1 = household is richer than the mean household asset value). To further improve statistical efficiency, we added school-level fixed effects (represented by $\Phi_{c}$) and computed robust standard errors (adjusted for clustering at the school level). Due to the presence of several observable and non-observable differences between the sampled schools, such as school policies and finances, we used school-level fixed effects to control for these variables and adjust for standard error. Within each school, students, parents, teachers, and principals tend to have similar characteristics, such as school performance. Thus, we uniquely coded each school and clustered according to school ID in our regression.

Third, to observe whether there is any interaction between a student’s math scores (which indicates academic performance) and certain control variables of interest (including ethnicity, gender, only child status, boarding status, parental migration status, and parent educational attainment) on depressive symptoms, we performed a heterogeneous analysis. For the heterogeneous analysis, we added interaction terms between the math score and variable of interest into the basic model. The new model is displayed in Equation (2):

(2) $$Y_{i}=\alpha +\beta_{1}score_{i}+\gamma x_{i}+\mu s_{i}+\rho \times score_{i}\times s_{i}+\Phi_{c}+\epsilon_{i},$$

with all the variables defined the same as those in Equation (1).

## 3. Results

### 3.1. Descriptive Statistics

Table 1 displays the descriptive statistics of student and household characteristics for the whole sample, the Han subsample, the Tibetan subsample, and Yi subsample. Panel A displays student characteristics. Within the whole sample, 16% were minorities (non-Han), 51% of students were female, and the age range was 7 to 19 years (mean = 11.35 years, SD = 1.05). Furthermore, 17% were only children (they had no siblings) and 9% were boarding students. For household characteristics, displayed in Panel B, 43% of students had fathers who had lived away from home for most of the previous semester, while 22% had mothers who had lived away from home for most of the previous semester. Regarding parent educational attainment, 18% of students had fathers with a high school or higher education and 20% had mothers with a high school or higher degree.

For descriptive statistics from the Han subsample, student characteristics reveal that 50% of Han students were female, and the average age of Han students was 11 years. Additionally, 18% were only children, and 5% were boarding students. Looking at household characteristics for the Han subsample, 43% of students had a father who migrated from home, and 23% had a mother who migrated. Regarding parental education, 20% of Han fathers had completed high school or above, and 22% of Han mothers had completed high school or above. In total, the sample included 7029 Han students (84.8% of the total sample).

For the Tibetan subsample, descriptive statistics of student characteristics show that 52% were female, and the average age was 11.5 years. Of the Tibetan students, 14% were only children, and 25% were boarding students. Regarding household characteristics of the Tibetan subsample, 33% and 22% of Tibetan students’ fathers and mothers, respectively, had migrated away from home. Tibetan parents, on average, held the same level of education, with 10% of both fathers and mothers having completed high school or above. The total number of Tibetan students sampled was 302 (3.6% of the total sample). 

Finally, when examining the descriptive statistics for the Yi subsample students, 54% of Yi students sampled were female, and the average age was 12 years. Additionally, 6% reported being the only child of their family, and 37% of Yi students were boarding students. For household characteristics, 49% of Yi fathers and 18% of Yi mothers had migrated from home, and 6% of fathers and 3% of mothers had completed high school or above. The total number of Yi students sampled was 979 (11.7% of the total sample). The remaining 0.9% of the sample includes 82 students who reported an ethnicity other than Han, Tibetan, or Yi. 

### 3.2. Prevalence of Depression among the Sample

Table 2 shows the prevalence of depression scores and depressive symptoms across the whole sample, Han subsample, Tibetan subsample, and Yi subsample, as well as comparisons between Han and Tibetan subsamples, and Han and Yi subsamples. For the whole sample, the average depression score was 5.95 points. Examining each ethnicity individually, Table 2 shows the mean score for Han subsample was 5.88 points (column 4 of row 1); the mean score for the Tibetan subsample was 5.96 points (column 6 of row 1); and the mean score for the Yi subsample was 6.46 points (column 8 of row 1). Comparing the mean depression scores of Han subsample and the minority subsamples, column 9 shows that there was no significant difference in depression scores between the Han and Tibetan subsamples. In contrast, column 10 reveals that there was a significant difference in the mean depression score between Han and Yi subsamples, with the mean Yi depression score 0.56 points higher the mean Han depression score (*p* < 0.001).

Regarding levels of depressive symptoms, across the whole sample, 19% was at risk of major depression (depression score ≥ 10); 2% was at risk of having severe depression (depression score ≥ 20). For Han students, 19% was at risk of having major depression and 2% was at risk of having severe depression. For Tibetan students, 18% showed risk of having major depression and 2% was at risk of having severe depression. Among Yi students, 22% was at risk of having major depression and 3% was at risk of having severe depression. Rows 9 and 10 show that there were no significant differences between any depressive symptom for Han and Tibetan subsamples, as well as Han and Yi subsamples.

### 3.3. Correlation between Academic Performance and Depressive Symptoms

Table 3 displays the correlations between academic performance and depressive symptoms in student and household characteristics. Math scores of the whole sample, as well as Han, Tibetan, and Yi subsamples are available in Table A1 of Appendix A. We identified significant correlations between depressive symptoms and specific student and household characteristics, including correlations between depressive symptoms and male students, younger students, minority students, and students with migrant parents. Specifically, being male was significantly correlated to moderately severe and severe depressive symptoms at the 5% significance level; being younger was significantly correlated to moderate and moderately severe depressive symptoms at the 1% level. Additionally, having a father who migrated away from home was significantly correlated to mild and moderate depressive symptoms at the 1% significance level, while having a mother who migrated away was significantly correlated with moderate depressive symptoms and major depression at the 5% significance level.

Using school-level fixed effects to control for student and household characteristics (Equation (1)), the results showed that students with higher math scores were at a significantly lower risk of depressive symptoms than students with lower math scores (Table 3). Specifically, an increase in math score by 1 SD was linked to a decrease of 0.47 points for the depression score (column 1 of row 1, significant at the 1% level). Furthermore, the results demonstrate that academic performance and risk of depression are significantly correlated. Lower risk of depression was reported among students with higher math scores, with a higher math score linked to a 4-percentage point decrease in the risk of major depression and a 6-percentage point decrease in the risk of moderate depression (row 1 columns 3 and 6, significant at the 1% level).

### 3.4. Heterogeneous Analysis

Table 4, Table 5, Table 6 and Table 7 examine individual subgroups (ethnicity; gender, boarding status, and only child status; parental migration status; and parent educational attainment, respectively) and their individual associations of academic performance and risk of depression, after adjusting for potential confounders.

#### 3.4.1. Ethnicity

Table 4 shows the correlations of academic performance and depressive symptoms for the ethnicity subgroups. Academic performance and risk of depression were negatively correlated in with both Han and minority (Yi and Tibetan) students. These results indicate that the risk of depression was higher among poor-performing minority and Han students than it was for students who performed better in school (row 1 and row 4, respectively). Specifically, a 1 SD increase in math score for minority students correlated to a 3% decrease in the risk of mild depression, an 8% decrease in risk of moderate depression, and a 4% decrease in the risk of major depression. Similarly, a 1 SD increase in math score for Han students was associated with a 3% decrease the risk of mild depression, 6% decrease in the risk of moderate depression, 2% decrease in risk of moderately severe depression, and 4% decrease in risk of major depression. Lastly, neither group (Han nor minority) exhibited any significant heterogeneous differences regarding the correlation between the academic performance and depressive symptoms.

#### 3.4.2. Gender, Boarding Student, and Only Child Status

Table 5 examines gender, boarding student, and only child subgroups and prevalent associations between academic performance and risk of depression. Panel A displays the gender subgroup. Academic performance was negatively correlated for male and female genders, as was the risk of depression for both genders. For male students, row 1 shows that a 1 SD increase in math score was significantly associated with a 3% decrease in mild depressive symptoms, a 5% decrease in moderate depressive symptoms, a 2% decrease in moderately severe depressive symptoms, and 3% decrease in major depression (significant at the 1% level). For female students, row 4 shows that a 1 SD increase in math score was associated with 3% decrease in mild depressive symptoms, 7% decrease in moderate depressive symptoms, 2% decrease in moderately severe depressive symptoms, and 4% decrease in major depression (significant at the 1% level). Columns 4 and 5 of row 3 indicate that the risks of moderately severe depression and severe depression were significantly higher for poor-performing male students than poor-performing female students (2%, and 1%, respectively, significant at the 5% level).

Panel B displays links between academic performance and depressive symptoms for boarding students and non-boarding students. For non-boarding students, row 5 shows that a 1 SD increase in math score was significantly associated with a 3% decrease in mild depressive symptoms, a 6% decrease in moderate depressive symptoms, a 2% decrease in moderately severe depressive symptoms, and a 4% decrease in major depression (significant at the 5% significance level). Row 8 shows that among students who boarded last semester, a 1 SD increase in math score was significantly associated with a 6% decrease in moderate depressive symptoms and 3% decrease in major depression (significant at the 1% level). In addition, column 1 of row 7 indicates that the risks of depression, with an increase of 1 SD for the math score, was 0.3 point greater among non-boarding students than boarding students (significant at the 5% level).

Panel C displays academic performance association with depression for the only child subgroup. Students with and without siblings were at higher risk of depressive symptoms across most depression levels (row 9 and row 12, respectively) than those who performed well. In row 9, we see that among non-only child students, a 1 SD increase in math score was significantly associated with a 3% decrease in mild depressive symptoms, a 6% decrease in moderate depressive symptoms, a 2% decrease in moderately severe depressive symptoms, and a decrease of 4% in risk of major depression (significant at the 1% level). For students with siblings, a 1 SD increase in math score was significantly associated with a 4% decrease in mild depressive symptoms, a 6% decrease in moderate depressive symptoms (at the 1% significance level), and a 3% decrease in major depression (row 12, at the 5% significance level). However, neither group (only child and non-only child) exhibited any significant heterogeneous differences regarding the correlation between the academic performance and depressive symptoms. Seen in row 11, there were no significant differences between correlations of academic performance and risk of depressive symptoms. Therefore, while an only child student might have a lower risk of developing major depression than a non-only child student (column 6 of row 10), their risk of depressive symptoms was not more strongly associated with poor academic performance than the risk of their non-only child peers.

#### 3.4.3. Parental Migration Status

The data in Table 6 show correlations between academic performance and depressive symptoms for students with and without migrant parents. Panel A shows the results for migrant fathers and Panel B displays the results for migrant mothers. For students without migrant fathers, a 1 SD increase in math score significantly corresponded to a 3% decrease in mild depressive symptoms, a 5% decrease in moderate depressive symptoms, a 2% decrease in moderately severe depressive symptoms, and a 4% decrease in major depression (significant at the 5% level). For students with migrant fathers, a 1 SD increase in math score was significantly associated with a 3% decrease in mild depressive symptoms, a 7% decrease in moderate depressive symptoms, a 2% decrease in moderately severe depressive symptoms, and a 4% decrease in major depression (significant at the 5% level). Across all depression levels, there was no significant heterogeneity between students with or without migrant fathers regarding the correlation between the academic performance and depressive symptoms. 

Similarly, Panel B indicates that better academic performance decreased the risk of depression among students with and without migrant mothers. For students without migrant mothers, a 1 SD increase in math score was significantly associated with a 3% decrease in mild depressive symptoms, a 6% decrease in moderate depressive symptoms, a 2% decrease in moderately severe depressive symptoms, and a 4% decrease in major depression (at the 5% significance level). For students with migrant mothers, a 1 SD increase in math score was associated with decreases in mild depressive symptoms and moderately severe depressive symptoms by 3% (at the 1% significance level), as well as decreases in moderate depressive symptoms by 7% and major depression by 4% (at the 5% significance level). Lastly, there was no significant heterogeneity across all depression levels with and without migrant mothers regarding the correlation between the academic performance and depressive symptoms.

#### 3.4.4. Parent Educational Attainment

Table 7 compares the correlations of academic performance and depressive symptoms between students of parents with different educational attainment levels, separately reporting results for fathers (Panel A) and mothers (Panel B). Panel A displays that for students with fathers who did not complete high school, a 1 SD increase in math score was significantly associated with a 3% decrease in mild depressive symptoms, a 6% decrease in moderate depressive symptoms, a 2% decrease in moderately severe depressive symptoms, and a 4% decrease in major depressive symptoms (significant at the 5% level). For students of fathers who had completed high school or above, a 1 SD increase in math score was significantly associated with 3% decreases in both moderate and moderately severe depressive symptoms (at the 1% significance level) and a 3% decrease in major depression (at the 5% significance level). There was no significant heterogeneity across all depression levels between fathers with different educational attainment levels regarding the correlation between the academic performance and depressive symptoms.

Likewise, a mother’s educational attainment appears to be a significant link for risk of depression. Students with lower math scores whose mothers had completed high school or above were at the greatest risk of depression. Specifically, an increase in math score by 1 SD for students with mothers who completed high school or above was correlated with a 4% decrease in risk of mild depression, a 6% decrease in risk of moderate depression, a 5% decrease in moderately severe depression, and a 5% decrease in major depressive symptoms. As for students with mothers did not complete high school, a 1 SD increase in math score was correlated to a 3% decrease in mild depression, a 6% decrease in moderate depression, a 1% decrease in moderately severe depression, and a 4% decrease in major depressive symptoms. Moreover, the risk of moderately severe depression was 4% higher for poor-performing students (1 SD decrease in math score) whose mothers completed high school or above than it was for students whose mothers did not complete high school (column 4 of row 7, significant at the 5% level).

## 4. Discussion

In this paper, we investigated the rates of depressive symptoms among rural Han and minority primary school students in Southwestern China and the links between academic performance and depression in subgroups. Within a sample of 8392 4th, 5th, and 6th graders, the risk of major depression is 19%, the risk of severe depression is 2%, and 5.95 is the average depression score. We found that the prevalence of depression is higher among minority students than Han students, with students of Yi ethnicity at greatest risk of major depression (22%) and severe depression (3%). In addition, poor academic performance, gender, age, and parent migration status were all significantly associated with the risk of depression. Furthermore, our heterogeneous analysis suggests that poor performing students who are male, are not boarding students, and whose mothers are more highly educated, are the subgroups at significantly higher risks of depression, with risk of depression increasing between 1–7% given a 1 SD decrease in academic performance.

Compared to the international literature, our results show that students from this study have similar and even higher rates of depression, both when using the same PHQ-8 scale, as well as when using the PHQ-9 scale. The PHQ-9 scale includes the same cutoff scores and questions as the PHQ-8 scale, except for one question about self-harm; moreover, the PHQ-9 scale has been validated as a comparison tool to the scores from the PHQ-8 series [48,49,50]. Given that no previous studies in rural China have used the PHQ-8, in comparison to studies of Chinese child and adolescent samples that used the PHQ-9, our sample reports higher rates of mild depression, moderate depression, moderately severe depression than similarly aged, non-minority group samples [40]. Though we cannot directly compare our results to studies that used different instruments to measure depressive symptoms, a systematic review and meta-analysis from 2020 on the prevalence of depressive symptoms in primary school students in China, with a sample of 27 studies that included 42,374 subjects, found that the pooled prevalence was 17.2% (95% CI: 14.3–20.5%) [13]. This indicates that beyond what instrument is used to measure depression, childhood and adolescent depression is a serious health concern in China, and requires greater attention from policymakers and researchers, alike.

Next, compared to international studies from other LMICs that use the PHQ-8, our sample reports similar levels of depressive symptoms across most severity levels. According to previous studies in other LMICs, the rate of risk of major depression among children and adolescents ranges from 7.6 to 28.1% [32,51,52]. On one hand, our sample reports even higher rates depressive symptoms than their counterparts in India [51], but slightly lower rates than their counterparts in Kenya and Nigeria [32,52]. On the other hand, while children and adolescents from rural China are at greater risk of mild and moderate depressive symptoms than their counterparts in western and more developed countries, like Norway and the United States; however, the rates of severe depressive symptoms are approximately the same [53,54]. Overall, our findings are consistent with the international literature, which suggests that depression is more prevalent in LMICs than in higher-income countries [55,56].

When comparing the prevalence of depression between rural Han and rural minority students, our results indicate that certain minority students are at greater risk of depression than Han students. Specifically, students who are Yi, one of the largest minority populations in China with a population of roughly 9 million people [26], are at the highest risk of major depression (22%) and severe depression (3%). In contrast, Han students have a risk of major depression of 19% and a risk of severe depression of 2%, which are similar to the risk of major depression (18%) and risk of severe depression (2%) among Tibetan students in our sample. Similar to our findings, Zhou et al. [8], who surveyed 2679 children aged 10–15 years old in China (using the CES-D), found a higher risk of depression among non-Han rural students than rural Han students. These results are crucial for developing interventions and policies that aim to treat mental health among children and adolescents in rural China, as it identifies several vulnerable and at-risk groups.

Moreover, our data show significant links between poor academic performance and risk of depression among rural Chinese youth. Increased academic performance scores (by 1 SD) were associated with a 3% decrease in mild depression, 6% decrease in risk of moderate depression, 2% decrease for moderately severe depression, and 4% decrease for major depression. While these decreases may seem small, those percent increases may determine a student’s depressive symptoms changing from moderate to mild, or severe to moderately severe, and should thus be noted for future research and practitioners studying mental illness in rural China. Perhaps stress from academics underlies the association between academic performance and mental health, as China’s education system and prioritization of examination may create a more prevalent environment of competition and anxiety for students. As Ang et al. [57] reported, in a sample of 1108 Asian adolescents in Singapore, academic stress is linked to depression. The same is true internationally, from Asia to Europe [4,5]. Moreover, academic stress can be compounded by a myriad of external factors, including social anxieties connected to academics [4]. For example, in China student exam scores are posted for all students to see. For poor-performing students, having low grades on full display may be an anxiety-producing tradition, which could compound any symptoms of depression.

Regardless of ethnicity, rural students are all vulnerable to developing depression; however, when struggling with academics, specific subgroups within each ethnic minority group (Han, Yi, Tibetan) are more likely to be at risk of depression. First, we identified that despite rural minority students reporting higher rates of depressive symptoms than their Han peers, both rural Han and minority students were at similar rates of risk of developing depressive symptoms if they performed poorly in school. In other words, poor academic performance did not disproportionally affect the rate of depressive symptoms between rural Han and minority students.

Second, despite finding that the risk of depression is not significantly different for male and female students, our evidence does suggest that the risk of developing depression might be greater for poor-performing male students than it is for poor-performing female students. One possible reason is that male students may experience more school-related stress than female students. According to previous study, male primary school students tend to experience stress and depressive symptoms surrounding school settings and activities, whereas female students experience stress and depressive symptoms around personal relationships [58]. This indicates that male students who struggle in primary school might be a target group for future interventions that focus on treating mental health issues.

In addition, the risk of depression is greater for poor-performing students if they live at home. According to a previous study examining the effects of peer support on depression among boarding students in rural China, social support from peers may help boarding students relieve stress and depressive feelings associated with negative life events, which includes negative events related to academics [59]. Thus, perhaps non-boarding students lack the social bonds and support boarding students share, which ultimately may influence the differences in risk of depression between boarding and non-boarding students. While the details of these underlying factors fall outside the scope of our study, such findings open the door for future research on school and mental health.

Parent educational attainment plays a significant role in the link between academic performance and risk of depression—specifically, a mother’s educational attainment. First, we found no evidence of greater risk of depression among poor-performing students whose fathers are less educated are at greater risk of depression than poor-performing students whose fathers are more educated. In contrast, the risk of moderately severe depression is higher among poor-performing students whose mothers completed high school or above. One plausible explanation is the relation between academic stress and family expectations common in East Asian countries [60,61]. Furthermore, research shows that in western China, a mother’s involvement in her child’s education is greater than a father’s involvement, which may be related to mother’s parenting behavior being more authoritative than a father’s [62]. Based on findings from these studies, children and adolescents may feel more academic stress from the expectations of their mothers than fathers, which may then manifest in more depressive symptoms being linked to mother characteristics. Previous research has found links between mothers’ emotions and criticism and their child’s depressive symptoms [63,64], which supports our findings.

Finally, while we found significant correlations between depression and student characteristics including age, being an only child, and having migrant parents, our results do not indicate that the rates of depression among poor-performing students are associated with these same characteristics. In other words, there are no significant associations between poor academic performance and depression among younger children, only children, or children of migrant parents. Despite the lack of significance, this finding is still crucial as it highlights how only children, students of migrant parents, and younger students—regardless of how they perform in school—are vulnerable to depressive symptoms.

We acknowledge several limitations of our study. First, due to our study using cross sectional data, we cannot identify causation. Thus, in the interpretation of our results, we must be careful to only interpret these links as correlative. This limitation is important to note for future lines of research that aim to either confirm or challenge such correlations or identify causal relationships between variables and depressive symptoms. Second, by collecting data in counties that had low levels of economic development and income, we did not collect a nationally representative sample. China’s demographic distribution is highly varied across the country, varying in rural and urban areas, socioeconomic status, and economic levels, and so on. Thus, the findings of this study cannot be broadly applied to the entirety of the country, but rather to similar samples located in rural areas of western China. Future studies might look to investigate other similar samples in different areas of China or broaden this study to samples across the entire country. Third, we did not apply the counterbalancing technique with respect to the order of administration of the evaluation instruments, which should be noted as an important bias to take into consideration of our results. However, despite not including this technique, we used standardized evaluation instruments, and administered them in a standardized way for all participants. Fourth, in measuring depressive symptoms, levels of severity (major depression, severe depression, etc.) were established using only cut-off points from a self-reported measure. Given the nature of self-reported measures in any study introducing a level of reporter bias, it may be important for future research to use clinical diagnosis through clinical interviews rather than self-reported measures of depressive symptoms for measuring prevalence and risk of depression, among any sample.

## 5. Conclusions

Our findings indicate that the risk of depression among primary school students in rural China is a pressing issue that requires further attention, from both researchers and policymakers. For researchers, these findings highlight specific group characteristics of students from rural China that make them more susceptible to depressive symptoms, especially with the interaction of poor academic performance. Future research could extend these findings and original research questions to broader samples across China, including rural communities beyond Southwestern China. In collecting a full national sample, broader findings about the state of childhood and adolescent mental health in China could be determined, and thus, more children and adolescents could be supported. For policymakers, the findings of this study are equally important, especially as the effects of the COVID-19 pandemic have influenced mental health outcomes among the youth around the globe. By understanding what communities and groups of students are more commonly affected by mental illnesses, policymakers could allocate a greater number of resources and support to particularly vulnerable subgroups. Ultimately, this paper makes a substantial contribution to the body of literature on childhood and adolescent mental health in LMICs, with significant findings on links between academic performance and depressive symptoms in a large sample of an understudied yet vulnerable population: rural minority children. In conclusion, poor academic performance is correlated to an increased risk of depression among rural Chinese children and adolescents. To ensure the healthy futures of the youth in China, both school- and home-based interventions that prevent and treat depression among children and adolescents, and interventions that provide emotional support to children struggling academically need to be implemented.

## Figures and Tables

**Table 1:** Descriptive statistics of student and household characteristics.

Variables/Groups		Whole Sample (N = 8392)		Han (N = 7029)		Tibetan (N = 302)		Yi (N = 979)	
		Mean/Percent	SD	Mean/Percent	SD	Mean/Percent	SD	Mean/Percent	SD
		(1)	(2)	(3)	(4)	(5)	(6)	(7)	(8)
Student characteristics (Panel A)									
(1)	Ethnicity (1 = Han)	0.84	0.37	-	-	-	-	-	-
(2)	Female (1 = yes)	0.51	0.50	0.50	0.50	0.52	0.50	0.54	0.50
(3)	Age (years)	11.35	1.05	11.26	0.97	11.53	1.13	11.95	1.37
(4)	Only child (1 = yes)	0.17	0.37	0.18	0.39	0.14	0.34	0.06	0.23
(5)	Boarding status (1 = yes)	0.09	0.29	0.05	0.22	0.25	0.43	0.37	0.48
Household characteristics (Panel B)									
(6)	Paternal migration (1 = yes)	0.43	0.49	0.43	0.50	0.33	0.47	0.39	0.49
(7)	Maternal migration (1 = yes)	0.22	0.42	0.23	0.42	0.22	0.41	0.18	0.39
(8)	Father completed high school or above (1 = yes)	0.18	0.38	0.20	0.40	0.10	0.30	0.06	0.24
(9)	Mother completed high school or above (1 = yes)	0.20	0.40	0.22	0.42	0.10	0.30	0.03	0.18
(10)	Asset index (PCA score)	0.01	1.31	0.16	1.28	−0.16	1.26	−1.03	1.03

The whole sample number (8392) includes all students who were Han, Tibetan, Yi, and other minority ethnicities; “SD” means standard deviation.

**Table 2:** Independent sample *t*-test results of depressive symptoms differences between Han, Tibetan and Yi subsamples.

Variables/Groups		Whole Sample (N = 8392)		Han (N = 7029)		Tibetan (N = 302)		Yi (N = 979)		Han vs. Tibetan		Han vs. Yi	
		Obs	Mean (SD)	Obs	Mean (SD)	Obs	Mean (SD)	Obs	Mean (SD)	Difference	*p*-Value	Difference	*p*-Value
		(1)	(2)	(3)	(4)	(5)	(6)	(7)	(8)	(9) = (4) − (6)	(9)	(10) = (4) − (8)	(10)
(1)	Depression score	8392	5.95	7029	5.88	302	5.96	979	6.46	−0.08	0.749	−0.56 **	<0.001
			(4.27)		(4.26)		(4.30)		(4.30)	(0.25)		(0.15)	
(2)	Mild depressive symptoms (5 ≤ Depression score ≤ 9; 1 = yes)	6804	0.48	5732	0.47	247	0.49	758	0.51	−0.02	0.538	−0.04 *	0.040
			(0.50)		(0.50)		(0.50)		(0.50)	(0.03)		(0.02)	
(3)	Moderate depressive symptoms (10 ≤ Depression score ≤ 14; 1 = yes)	4818	0.26	4064	0.26	170	0.26	541	0.32	0.00	1.000	−0.06 **	0.003
			(0.44)		(0.44)		(0.44)		(0.47)	(0.03)		(0.02)	
(4)	Moderately severe depressive symptoms (15 ≤ Depression score ≤ 19; 1 = yes)	3786	0.06	3219	0.06	132	0.05	403	0.09	0.01	0.638	−0.03 *	0.020
			(0.25)		(0.24)		(0.22)		(0.28)	(0.02)		(0.01)	
(5)	Severe depressive symptoms (Depression score ≥ 20; 1 = yes)	3610	0.02	3070	0.02	128	0.02	380	0.03	0.00	1.000	−0.01	0.180
			(0.13)		(0.13)		(0.15)		(0.18)	(0.01)		(0.01)	
(6)	Major depression (Depression score ≥ 10; 1 = Yes)	8392	0.19	7029	0.19	302	0.18	979	0.22	0.01	0.660	−0.03 *	0.026
			(0.39)		(0.39)		(0.39)		(0.42)	(0.02)		(0.01)	

The whole sample number (8392) includes all students who were Han, Tibetan, Yi, and other minority ethnicities; “Obs” means observations; “SD” means standard deviation. ** *p* < 0.01, * *p* < 0.05.

**Table 3:** Multivariate multiple linear regressions between academic performance and depressive symptoms across individual and family characteristics.

Variables		Depression Score	Mild Depressive Symptoms	Moderate Depressive Symptoms	Moderately Severe Depressive Symptoms	Severe Depressive Symptoms	Major Depression
		(1)	(2)	(3)	(4)	(5)	(6)
(1)	Math score (SD)	−0.47 **	−0.03 **	−0.06 **	−0.02 **	−0.00	−0.04 **
		(0.05)	(0.01)	(0.01)	(0.00)	(0.00)	(0.00)
(2)	Ethnicity (1 = Han)	0.08	−0.03	0.01	0.02	−0.02 *	0.03
		(0.16)	(0.02)	(0.02)	(0.02)	(0.01)	(0.02)
(3)	Female (1 = yes)	−0.06	0.03	0.01	−0.02 *	−0.01 *	−0.01
		(0.12)	(0.01)	(0.01)	(0.01)	(0.01)	(0.01)
(4)	Age (years)	−0.05	−0.01	−0.02 **	0.01 **	0.01	−0.00
		(0.06)	(0.01)	(0.01)	(0.00)	(0.00)	(0.01)
(5)	Only child (1 = yes)	−0.02	0.01	−0.03	−0.00	0.01	−0.02
		(0.12)	(0.02)	(0.01)	(0.01)	(0.01)	(0.01)
(6)	Boarding status (1 = yes)	0.05	0.04	0.03	−0.03	0.01	−0.00
		(0.20)	(0.03)	(0.03)	(0.02)	(0.01)	(0.02)
(7)	Father completed high school or above (1 = yes)	0.03	0.00	−0.01	0.01	−0.00	−0.01
		(0.15)	(0.02)	(0.02)	(0.01)	(0.01)	(0.01)
(8)	Mother completed high school or above (1 = yes)	0.08	0.01	0.00	−0.00	0.00	0.00
		(0.13)	(0.02)	(0.02)	(0.01)	(0.01)	(0.01)
(9)	Paternal migration (1 = yes)	0.24 *	0.04 **	0.04 **	0.00	0.00	0.01
		(0.10)	(0.01)	(0.01)	(0.01)	(0.01)	(0.01)
(10)	Maternal migration (1 = yes)	0.43 **	0.02	0.04 *	0.01	0.01	0.03 *
		(0.11)	(0.02)	(0.02)	(0.01)	(0.01)	(0.01)
(11)	Richer household (1 = yes)	0.04	0.01	0.01	0.00	0.00	0.00
		(0.04)	(0.01)	(0.01)	(0.00)	(0.00)	(0.00)
(12)	School fixed effects	Yes	Yes	Yes	Yes	Yes	Yes
(13)	Observations	8392	6804	4818	3786	3610	8392
(14)	R-squared	0.06	0.04	0.08	0.06	0.05	0.04

All regressions control for student, household characteristics and strata fixed effects; “SD” means standard deviation; the numbers in parentheses represent standard errors; robust standard error are clustered at the school level; * indicates significant at 5%; ** indicates significant at 1%.

**Table 4:** Multivariate multiple linear regressions results showing heterogeneous effects of academic performance on depressive symptoms between different ethnicities.

Variables		Depression Score	Mild Depressive Symptoms	Moderate Depressive Symptoms	Moderately Severe Depressive Symptoms	Severe Depressive Symptoms	Major Depression
		(1)	(2)	(3)	(4)	(5)	(6)
	Ethnicity						
(1)	Math score (SD) ^a^	−0.38 **	−0.03 *	−0.08 **	−0.01	0.00	−0.04 **
		(0.09)	(0.01)	(0.02)	(0.01)	(0.01)	(0.01)
(2)	Ethnicity (1 = Han)	0.06	−0.03	0.02	0.02	−0.02	0.03
		(0.15)	(0.02)	(0.02)	(0.02)	(0.01)	(0.02)
(3)	Ethnicity × Math score	−0.12	0.01	0.02	−0.01	−0.00	0.00
		(0.12)	(0.02)	(0.02)	(0.01)	(0.01)	(0.01)
(4)	Ethnicity = (1) + (3) ^b^	−0.49 **	−0.03 **	−0.06 **	−0.02 **	−0.00	−0.04 **
		(0.06)	(0.01)	(0.01)	(0.01)	(0.00)	(0.01)

All regressions control for student, household characteristics and strata fixed effects; “SD” means standard deviation; the numbers in parentheses represent standard errors; robust standard error are clustered at the school level; * indicates significant at 5%; ** indicates significant at 1%. ^a^ Math score and depression association for students who minorities; ^b^ math score and depression association for students who are Han.

**Table 5:** Multivariate multiple linear regressions results showing heterogeneous effects of academic performance on depressive symptoms between different student characteristics.

Variables		Depression Score	Mild Depressive Symptoms	Moderate Depressive Symptoms	Moderately Severe Depressive Symptoms	Severe Depressive Symptoms	Major Depression
		(1)	(2)	(3)	(4)	(5)	(6)
	Gender (Panel A)						
(1)	Math score (SD) ^a^	−0.43 **	−0.03 **	−0.05 **	−0.02 **	−0.00	−0.03 **
		(0.07)	(0.01)	(0.01)	(0.01)	(0.00)	(0.01)
(2)	Female (1 = yes)	−0.08	0.00	−0.01	0.00	−0.00	−0.01
		(0.09)	(0.01)	(0.01)	(0.01)	(0.01)	(0.01)
(3)	Female × Math score	−0.06	0.03	0.01	−0.02 *	−0.01 *	−0.01
		(0.12)	(0.01)	(0.01)	(0.01)	(0.01)	(0.01)
(4)	Female = (1) + (3) ^b^	−0.51 **	−0.03 **	−0.07 **	−0.02 **	0.00	−0.04 **
		(0.07)	(0.01)	(0.01)	(0.01)	(0.00)	(0.01)
	Whether student is a boarding student (Panel B)						
(5)	Math score (SD) ^c^	−0.49 **	−0.03 **	−0.06 **	−0.02 **	−0.00	−0.04 **
		(0.06)	(0.01)	(0.01)	(0.00)	(0.00)	(0.00)
(6)	Boarding status (1 = yes)	−0.02	0.04	0.03	−0.03	0	−0.00
		(0.21)	(0.03)	(0.03)	(0.02)	(0.01)	(0.02)
(7)	Boarding status × Math score	0.30 *	0.01	0.00	0.02 *	0.01	0.01
		(0.12)	(0.02)	(0.02)	(0.01)	(0.01)	(0.01)
(8)	Boarding status = (5) + (7) ^d^	−0.20 *	−0.02	−0.06 *	0.00	0.00	−0.03 *
		(0.11)	(0.02)	(0.02)	(0.01)	(0.01)	(0.01)
	Whether student is an only child (Panel C)						
(9)	Math score (SD) ^e^	−0.47 **	−0.03 **	−0.06 **	−0.02 **	−0.00	−0.04 **
		(0.05)	(0.01)	(0.01)	(0.00)	(0.00)	(0.00)
(10)	Only child (1 = yes)	−0.02	0.01	−0.03	−0.00	0.01	−0.02
		−0.12	(0.02)	(0.01)	(0.01)	(0.01)	(0.01)
(11)	Only child × Math score	0.00	−0.01	−0.00	0.01	0.00	0.01
		(0.11)	(0.01)	(0.02)	(0.01)	(0.01)	(0.01)
(12)	Only child = (9) + (11) ^f^	−0.47 **	−0.04 **	−0.06 **	−0.01	−0.00	−0.03 *
		(0.11)	(0.01)	(0.02)	(0.01)	(0.01)	(0.01)

All regressions control for student, household characteristics and strata fixed effects; “SD” means standard deviation; the numbers in parentheses represent standard errors; robust standard error are clustered at the school level; * indicates significant at 5%; ** indicates significant at 1%. ^a^ Math score and depression association for male gender; ^b^ math score and depression association for female gender; ^c^ Math score and depression association for non-boarding students; ^d^ math score and depression association for boarding students; ^e^ math score and depression association for non-only children; ^f^ math score and depression association for students who are only children.

**Table 6:** Multivariate multiple linear regressions results showing heterogeneous effects of academic performance on depressive symptoms between different parental migration status.

Variables		Depression Score	Mild Depressive Symptoms	Moderate Depressive Symptoms	Moderately Severe Depressive Symptoms	Severe Depressive Symptoms	Major Depression
		(1)	(2)	(3)	(4)	(5)	(6)
	Father migration status (Panel A)						
(1)	Math score (SD) ^a^	−0.45 **	−0.03 **	−0.05 **	−0.02 **	−0.00	−0.04 **
		(0.06)	(0.01)	(0.01)	(0.01)	(0.00)	(0.00)
(2)	Paternal migration (1 = yes)	0.24 *	0.04 **	0.04 **	0.00	0.01	0.01
		(0.10)	(0.01)	(0.01)	(0.01)	(0.01)	(0.01)
(3)	Paternal migration × Math score	−0.06	−0.00	−0.01	−0.00	−0.01	−0.01
		(0.09)	(0.01)	(0.01)	(0.01)	(0.01)	(0.01)
(4)	Paternal migration = (1) + (3) ^b^	−0.51 **	−0.03 **	−0.07 **	−0.02 **	−0.01	−0.04 **
		(0.08)	(0.01)	(0.01)	(0.01)	(0.00)	(0.01)
	Mother migration status (Panel B)						
(5)	Math score (SD) ^c^	−0.45 **	−0.03 **	−0.06 **	−0.02 **	−0.00	−0.04 **
		(0.05)	(0.01)	(0.01)	(0.00)	(0.00)	(0.00)
(6)	Maternal migration (1 = yes)	0.43 **	0.02	0.04 *	0.01	0.01	0.03 *
		(0.11)	(0.02)	(0.02)	(0.01)	(0.01)	(0.01)
(7)	Maternal migration × Math score	−0.10	0.00	−0.01	−0.01	0.00	−0.01
		(0.11)	(0.02)	(0.02)	(0.01)	(0.01)	(0.01)
(8)	Maternal migration = (5) + (7) ^d^	−0.55 **	−0.03 *	−0.07 **	−0.03 *	−0.00	−0.04 **
		(0.10)	(0.01)	(0.02)	(0.01)	(0.01)	(0.01)

All regressions control for student, household characteristics and strata fixed effects; “SD” means standard deviation; the numbers in parentheses represent standard errors; robust standard error are clustered at the school level; * indicates significant at 5%; ** indicates significant at 1%. ^a^ Math score and depression association for students of non-migrant fathers; ^b^ math score and depression association for students of migrant fathers; ^c^ math score and depression association for students of non-migrant mothers; ^d^ math score and depression association for students of migrant mothers.

**Table 7:** Multivariate multiple linear regressions results showing heterogeneous effects of academic performance on depressive symptoms between different parent educational attainment.

Variables		Depression Score	Mild Depressive Symptoms	Moderate Depressive Symptoms	Moderately Severe Depressive Symptoms	Severe Depressive Symptoms	Major Depression
		(1)	(2)	(3)	(4)	(5)	(6)
	Father educational attainment (Panel A)						
(1)	Math score (SD) ^a^	−0.48 **	−0.03 **	−0.06 **	−0.02 **	−0.00	−0.04 **
		(0.05)	(0.01)	(0.01)	(0.00)	(0.00)	(0.00)
(2)	Father completed high school or above (1 = yes)	0.03	−0.00	−0.02	0.02	−0.00	−0.01
		(0.16)	(0.02)	(0.02)	(0.01)	(0.01)	(0.01)
(3)	Father education × Math score	0.04	0.01	0.039	−0.01	−0.01	0.01
		(0.15)	(0.02)	(0.02)	(0.02)	(0.01)	(0.01)
(4)	Father education = (1) + (3) ^b^	−0.44 **	−0.02	−0.03 *	−0.03 *	−0.01	−0.03 **
		(0.14)	(0.02)	(0.01)	(0.01)	(0.01)	(0.01)
	Mother educational attainment (Panel B)						
(5)	Math score (SD) ^c^	−0.44 **	−0.03 **	−0.06 **	−0.01 *	−0.00	−0.04 **
		(0.05)	(0.01)	(0.01)	(0.00)	(0.00)	(0.00)
(6)	Mother completed high school or above (1 = yes)	0.10	0.01	0.00	0.01	0.00	0.00
		(0.13)	(0.02)	(0.02)	(0.01)	(0.01)	(0.01)
(7)	Mother education × Math score	−0.19	−0.01	−0.01	−0.04 *	−0.00	−0.01
		(0.12)	(0.02)	(0.02)	(0.02)	(0.01)	(0.01)
(8)	Mother education = (5) + (7) ^d^	−0.63 **	−0.04 *	−0.06 **	−0.05 **	−0.01	−0.05 **
		(0.12)	(0.02)	(0.02)	(0.01)	(0.01)	(0.01)

All regressions control for student, household characteristics and strata fixed effects; “SD” means standard deviation; the numbers in parentheses represent standard errors; robust standard errors are clustered at the school level; * indicates significant at 5%; ** indicates significant at 1%. ^a^ Math score and depression association for students of fathers who did not complete high school or above; ^b^ math score and depression association for students of fathers who completed high school or above; ^c^ math score and depression association for students of mothers who did not complete high school or above; ^d^ math score and depression association for students of mothers who completed high school or above.

## Data Availability

Data are available upon request.

## Appendix A

**Table A1:** Math score differences between Han, Tibetan, and Yi.

Variables/Groups	Whole Sample (N = 8392)		Han (N = 7029)		Tibetan (N = 302)		Yi (N = 979)		Han vs. Tibetan		Han vs. Yi	
	Obs	Mean (SD)	Obs	Mean (SD)	Obs	Mean (SD)	Obs	Mean (SD)	Difference	*p*-Value	Difference	*p*-Value
	(1)	(2)	(3)	(4)	(5)	(6)	(7)	(8)	(9) = (4) − (6)	(9)	(10) = (4) − (8)	(10)
Math score (SD)	8392	0.01	7029	0.05	302	−0.12	979	−0.22	0.17 **	0.003	0.27 **	<0.001
		(0.99)		(0.98)		(0.98)		(0.99)	(0.06)		(0.03)	

The whole sample number (8392) includes all students who were Han, Tibetan, Yi, and other minority ethnicities; “Obs” means observations; “SD” means standard deviation. ** *p* < 0.01.

## References

[1] Kieling C., Baker-Henningham H., Belfer M., Conti G., Ertem I., Omigbodun O., Rohde L.A., Srinath S., Ulkuer N., Rahman A. (2011). Child and Adolescent Mental Health Worldwide: Evidence for Action. Lancet.

[2] Foley D.L., Goldston D.B., Costello E.J., Angold A. (2006). Proximal Psychiatric Risk Factors for Suicidality in Youth: The Great Smoky Mountains Study. Arch. Gen. Psychiatry.

[3] Copeland W.E., Alaie I., Jonsson U., Shanahan L. (2021). Associations of Childhood and Adolescent Depression With Adult Psychiatric and Functional Outcomes. J. Am. Acad. Child Adolesc. Psychiatry.

[4] Arusha A.R., Biswas R.K. (2020). Prevalence of Stress, Anxiety and Depression Due to Examination in Bangladeshi Youths: A Pilot Study. Child. Youth Serv. Rev..

[5] Barker E.T., Howard A.L., Villemaire-Krajden R., Galambos N.L. (2018). The Rise and Fall of Depressive Symptoms and Academic Stress in Two Samples of University Students. J. Youth Adolesc..

[6] Jayanthi P., Thirunavukarasu M., Rajkumar R. (2015). Academic Stress and Depression among Adolescents: A Cross-Sectional Study. Indian Pediatr..

[7] Gao Y., Hu D., Peng E., Abbey C., Ma Y., Wu C.-I., Chang C.-Y., Hung W.-T., Rozelle S. (2020). Depressive Symptoms and the Link with Academic Performance among Rural Taiwanese Children. Int. J. Environ. Res. Public Health.

[8] Zhou M., Zhang G., Rozelle S., Kenny K., Xue H. (2018). Depressive Symptoms of Chinese Children: Prevalence and Correlated Factors among Subgroups. Int. J. Environ. Res. Public Health.

[9] Rao W.-W., Zhang J.-W., Zong Q.-Q., An F.-R., Ungvari G.S., Balbuena L., Yang F.-Y., Xiang Y.-T. (2019). Prevalence of Depressive Symptoms in Overweight and Obese Children and Adolescents in Mainland China: A Meta-Analysis of Comparative Studies and Epidemiological Surveys. J. Affect. Disord..

[10] Wang L., Feng Z., Yang G., Yang Y., Wang K., Dai Q., Zhao M., Hu C., Zhang R., Liu K. (2016). Depressive Symptoms among Children and Adolescents in Western China: An Epidemiological Survey of Prevalence and Correlates. Psychiatry Res..

[11] Li C., Wang H., Cao X., Gou M., Zhang Z. (2013). [Depressive symptoms and its related factors among primary and middle school students in an urban-rural-integrated area of Chongqing]. Wei Sheng Yan Jiu.

[12] Li J.-Y., Li J., Liang J.-H., Qian S., Jia R.-X., Wang Y.-Q., Xu Y. (2019). Depressive Symptoms Among Children and Adolescents in China: A Systematic Review and Meta-Analysis. Med. Sci. Monit..

[13] Xu D.-D., Rao W.-W., Cao X.-L., Wen S.-Y., An F.-R., Che W.-I., Bressington D.T., Cheung T., Ungvari G.S., Xiang Y.-T. (2020). Prevalence of Depressive Symptoms in Primary School Students in China: A Systematic Review and Meta-Analysis. J. Affect. Disord..

[14] Tan J.B., Yates S. (2011). Academic Expectations as Sources of Stress in Asian Students. Soc. Psychol. Educ..

[15] Sun J., Dunne M.P., Hou X., Xu A. (2011). Educational Stress Scale for Adolescents: Development, Validity, and Reliability With Chinese Students. J. Psychoeduc. Assess..

[16] Li H., Zhang Y. (2008). Factors Predicting Rural Chinese Adolescents’ Anxieties, Fears and Depression. Sch. Psychol. Int..

[17] Liu H., Shi Y., Auden E., Rozelle S. (2018). Anxiety in Rural Chinese Children and Adolescents: Comparisons across Provinces and among Subgroups. Int. J. Environ. Res. Public Health.

[18] Chen Q., Chen Y., Zhao Q. (2020). Impacts of Boarding on Primary School Students’ Mental Health Outcomes—Instrumental-Variable Evidence from Rural Northwestern China. Econ. Hum. Biol..

[19] Tang W., Hu T., Hu B., Jin C., Wang G., Xie C., Chen S., Xu J. (2020). Prevalence and Correlates of PTSD and Depressive Symptoms One Month after the Outbreak of the COVID-19 Epidemic in a Sample of Home-Quarantined Chinese University Students. J. Affect. Disord..

[20] Chalasani S. (2007). The Changing Relationship between Parents’ Education and Their Time with Children. Int. J. Time Use Res..

[21] Guryan J., Hurst E., Kearney M. (2008). Parental Education and Parental Time with Children. J. Econ. Perspect..

[22] Sonego M., Llácer A., Galán I., Simón F. (2013). The Influence of Parental Education on Child Mental Health in Spain. Qual. Life Res..

[23] Liu Y.-L. (2003). Parent–Child Interaction and Children’s Depression: The Relationships between Parent–Child Interaction and Children’s Depressive Symptoms in Taiwan. J. Adolesc..

[24] Lawson D.W., Mace R. (2010). Siblings and Childhood Mental Health: Evidence for a Later-Born Advantage. Soc. Sci. Med..

[25] Pedersen C.B., Mortensen P.B. (2004). Sibship Characteristics during Upbringing and Schizophrenia Risk. Am. J. Epidemiol..

[26] Liu A., Ye Z. (2020). China Statistical Yearbook-2020.

[27] Song K., Luo Z., Deng S., Zhou H. (2020). Ya’an Statistical Yearbook-2020.

[28] Kroenke K., Spitzer R.L., Williams J. (2001). The PHQ-9 Validity of a Brief Depression Severity Measure. J. Gen. Intern. Med..

[29] Kroenke K., Strine T., Spitzer R., Williams J., Berry J., Mokdad A. (2008). The PHQ-8 as a Measure of Current Depression in the General Population. J. Affect. Disord..

[30] Razykov I., Ziegelstein R.C., Whooley M.A., Thombs B.D. (2012). The PHQ-9 versus the PHQ-8—Is Item 9 Useful for Assessing Suicide Risk in Coronary Artery Disease Patients? Data from the Heart and Soul Study. J. Psychosom. Res..

[31] Campbell S., Osborn T.L. (2021). Adolescent Psychopathology and Psychological Wellbeing: A Network Analysis Approach. BMC Psychiatry.

[32] Osborn T.L., Venturo-Conerly K.E., Gan J., Rodriguez M., Alemu R.G., Roe E., Arango S., Wasil A.R., Campbell S., Weisz J.R. (2021). Depression and Anxiety Symptoms Amongst Kenyan Adolescents: Psychometric Properties, Prevalence, Sociodemographic Factors, and Psychological Wellbeing. PsyArXiv.

[33] López-Torres S., Pérez-Pedrogo C., Sánchez-Cardona I., Sánchez-Cesáreo M. (2019). Psychometric Properties of the PHQ-A among a Sample of Children and Adolescents in Puerto Rico. Curr. Psychol..

[34] Lu W., Bian Q., Song Y., Ren J., Xu X., Zhao M. (2015). Prevalence and Related Risk Factors of Anxiety and Depression among Chinese College Freshmen. J. Huazhong Univ. Sci. Technol. Med. Sci..

[35] Wang W., Bian Q., Zhao Y., Li X., Wang W., Du J., Zhang G., Zhou Q., Zhao M. (2014). Reliability and Validity of the Chinese Version of the Patient Health Questionnaire (PHQ-9) in the General Population. Gen. Hosp. Psychiatry.

[36] Sobowale K., Zhou N., Fan J., Liu N., Sherer R. (2014). Depression and Suicidal Ideation in Medical Students in China: A Call for Wellness Curricula. Int. J. Med. Educ..

[37] Zhou Y., Xu J., Rief W. (2020). Are Comparisons of Mental Disorders between Chinese and German Students Possible? An Examination of Measurement Invariance for the PHQ-15, PHQ-9 and GAD-7. BMC Psychiatry.

[38] Chen S., Chiu H., Xu B., Ma Y., Jin T., Wu M., Conwell Y. (2010). Reliability and Validity of the PHQ-9 for Screening Late-Life Depression in Chinese Primary Care. Int. J. Geriatr. Psychiatry.

[39] Tsai F.-J., Huang Y.-H., Liu H.-C., Huang K.-Y., Huang Y.-H., Liu S.-I. (2014). Patient Health Questionnaire for School-Based Depression Screening Among Chinese Adolescents. Pediatrics.

[40] Kong L.-Z., Lai J.-B., Hu S.-H. (2022). China Initiates Depression Screening in Children and Adolescents. Lancet Psychiatry.

[41] Leung D.Y.P., Mak Y.W., Leung S.F., Chiang V.C.L., Loke A.Y. (2020). Measurement Invariances of the PHQ-9 across Gender and Age Groups in Chinese Adolescents. Asia-Pac. Psychiatry.

[42] Chu J.H., Loyalka P., Chu J., Qu Q., Shi Y., Li G. (2015). The Impact of Teacher Credentials on Student Achievement in China. China Econ. Rev..

[43] Li L., Huang L., Shi Y., Luo R., Yang M., Rozelle S. (2018). Anemia and Student’s Educational Performance in Rural Central China: Prevalence, Correlates and Impacts. China Econ. Rev..

[44] Loyalka P., Popova A., Li G., Shi Z. (2019). Does Teacher Training Actually Work? Evidence from a Large-Scale Randomized Evaluation of a National Teacher Training Program. Am. Econ. J. Appl. Econ..

[45] Loyalka P., Sylvia S., Liu C., Chu J., Shi Y. (2019). Pay by Design: Teacher Performance Pay Design and the Distribution of Student Achievement. J. Labor Econ..

[46] Ma Y., Fairlie R.W., Loyalka P., Rozelle S. (2020). Isolating the “Tech” from EdTech: Experimental Evidence on Computer Assisted Learning in China.

[47] Kolenikov S., Angeles G. (2009). Socioeconomic Status Measurement with Discrete Proxy Variables: Is Principal Component Analysis a Reliable Answer?. Rev. Income Wealth.

[48] Shin C., Lee S.-H., Han K.-M., Yoon H.-K., Han C. (2019). Comparison of the Usefulness of the PHQ-8 and PHQ-9 for Screening for Major Depressive Disorder: Analysis of Psychiatric Outpatient Data. Psychiatry Investig..

[49] Wu Y., Levis B., Riehm K.E., Saadat N., Levis A.W., Azar M., Rice D.B., Boruff J., Cuijpers P., Gilbody S. (2020). Equivalency of the Diagnostic Accuracy of the PHQ-8 and PHQ-9: A Systematic Review and Individual Participant Data Meta-Analysis. Psychol. Med..

[50] Oncology Nursing Society (2010). Instructions for Patient Health Questionnaire (PHQ) and GAD-7 Measures.

[51] Singh M.M., Gupta M., Grover S. (2017). Prevalence & Factors Associated with Depression among Schoolgoing Adolescents in Chandigarh, North India. Indian J. Med. Res..

[52] Fatiregun A.A., Kumapayi T.E. (2014). Prevalence and Correlates of Depressive Symptoms among In-School Adolescents in a Rural District in Southwest Nigeria. J. Adolesc..

[53] Burdzovic Andreas J., Brunborg G.S. (2017). Depressive Symptomatology among Norwegian Adolescent Boys and Girls: The Patient Health Questionnaire-9 (PHQ-9) Psychometric Properties and Correlates. Front. Psychol..

[54] Richardson L.P., McCauley E., Grossman D.C., McCarty C.A., Richards J., Russo J.E., Rockhill C., Katon W. (2010). Evaluation of the Patient Health Questionnaire (PHQ-9) for Detecting Major Depression among Adolescents. Pediatrics.

[55] Lotfaliany M., Hoare E., Jacka F.N., Kowal P., Berk M., Mohebbi M. (2019). Variation in the Prevalence of Depression and Patterns of Association, Sociodemographic and Lifestyle Factors in Community-Dwelling Older Adults in Six Low- and Middle-Income Countries. J. Affect. Disord..

[56] Mukadam N., Sommerlad A., Huntley J., Livingston G. (2019). Population Attributable Fractions for Risk Factors for Dementia in Low-Income and Middle-Income Countries: An Analysis Using Cross-Sectional Survey Data. Lancet Glob. Health.

[57] Ang R.P., Huan V.S. (2006). Relationship between Academic Stress and Suicidal Ideation: Testing for Depression as a Mediator Using Multiple Regression. Child Psychiatry Hum. Dev..

[58] Bastin M., Mezulis A.H., Ahles J., Raes F., Bijttebier P. (2015). Moderating Effects of Brooding and Co-Rumination on the Relationship between Stress and Depressive Symptoms in Early Adolescence: A Multi-Wave Study. J. Abnorm. Child Psychol..

[59] Gao J.-L., Wang L.-H., Yin X.-Q., Hsieh H.-F., Rost D.H., Zimmerman M.A., Wang J.-L. (2021). The Promotive Effects of Peer Support and Active Coping in Relation to Negative Life Events and Depression in Chinese Adolescents at Boarding Schools. Curr. Psychol..

[60] Sun J., Dunne M.P., Hou X., Xu A. (2013). Educational Stress among Chinese Adolescents: Individual, Family, School and Peer Influences. Educ. Rev..

[61] Yoon E., Adams K., Clawson A., Chang H., Surya S., Jérémie-Brink G. (2017). East Asian Adolescents’ Ethnic Identity Development and Cultural Integration: A Qualitative Investigation. J. Couns. Psychol..

[62] Ho H.-Z., Chen W.-W., Tran C.N., Ko C.-T. (2010). Parental Involvement in Taiwanese Families: Father-Mother Differences. Child. Educ..

[63] Crugnola C.R., Ierardi E., Ferro V., Gallucci M., Parodi C., Astengo M. (2016). Mother-Infant Emotion Regulation at Three Months: The Role of Maternal Anxiety, Depression and Parenting Stress. Psychopathology.

[64] Muzik M., Rosenblum K. (2016). A Mental Health and Parenting Intervention for Adolescent and Young Adult Mothers and Their Infants. J. Depress. Anxiety.

